# A Combination of *Punica granatum* Fruit Rind and *Theobroma cacao* Seed Extracts Enhances Sexual Function in Aging Males in a Randomized, Double-blind, Placebo-controlled Study

**DOI:** 10.7150/ijms.99958

**Published:** 2025-01-01

**Authors:** Manoj Kumar Srivastava, Gaurav Singh, Raveendra Ramamurthy Kodur, Amulya Yalamanchi

**Affiliations:** 1 Department of General Medicine, Kashi Medicare, Varanasi-221001, Uttar Pradesh, India.; 2 Department of General Medicine, Upendra Medicare, Varanasi-221001, Uttar Pradesh, India.; 3 Department of Medicine, Sapthagiri Institute of Medical Sciences and Research Centre, Bengaluru-560090, Karnataka, India.; 4 Department of General Medicine, Yalamanchi Hospitals and Research Centre, Vijayawada-520002, Andhra Pradesh, India.

**Keywords:** Comprehensive safety, Derogatis Interview for Sexual Functioning-Male (DISF-SR-M), International Index of Erectile Function (IIEF), Testosterone, *Punica granatum*, *Theobroma cacao*

## Abstract

**Introduction:** LN18178 is a standardized, synergistic combination of *Punica granatum* fruit rind and *Theobroma cacao* seed extracts, which has been reported to increase serum testosterone levels in young and aging males.

**Methods:** The present 84-day randomized, double-blind, placebo-controlled study assessed the efficacy of LN18178 on the sexual function of aging male volunteers (age: 40-70 years; serum total testosterone: ≥ 300 ng/dL). The subjects with mild to moderate erectile dysfunction [5-item version of the International Index of Erectile Function (IIEF-5) scores 17-25] and low sexual desire (score < 3 on items 11 and 12 of IIEF) participated in this investigation. One hundred and twenty men were randomly allocated into either the LN18178 or placebo group (n=60); they took either 400 mg of LN18178 or a matched placebo capsule daily with breakfast.

**Results:** Post-trial, the LN18178-supplemented participants reported significant (P < 0.05) improvements in total and domain scores of the Derogatis Interview for Sexual Functioning-Self Reporting Male (DISF-SR-M) questionnaire, as well as substantial improvements in IIEF-5 (International Index of Erectile Function-5) and erection hardness scores (EHS). Comparative analysis also revealed significant improvements in the multi-dimensional fatigue inventory (MFI) and general health survey (GHS) scores. LN18178 supplementation substantially (P < 0.05) increased the six-minute walk distance and hand-grip strength compared to placebo. The participants' hemato-biochemical parameters, urinalysis, and vitals were within the normal range.

**Conclusion:** LN18178 enhances sexual function, libido and improves psychological well-being, as well as neuromotor function and general well-being in aging males. LN18178 supplementation is safe and well tolerated by the participants.

## Introduction

Advancing age in men declines endocrine functions that result in a complex and multifaceted array of reduced physiological and biochemical events, including psychological functions 1. Sexual desire and performance reduce with advancing age and cause dissatisfaction, impairing sexual health that negatively influences men's and their partner's quality of life and social functionality. According to the World Health Organization (WHO), “Good sexual and reproductive health is a state of complete physical, mental, and social well-being in all matters relating to the reproductive system.” 2. In men, among various biological and psychological factors, endocrine function plays a major role in regulating sexual wellness, including sexual function and performance 3.

Generally, testosterone levels in males fall by 0.4 to 2% per year after age 30 4. Testosterone is the major androgen responsible for the growth and development of the male reproductive system, which helps maintain typical male sexual characteristics, including sexual function, desire, performance, vigor, healthy sperm profile, bone and muscle mass, metabolic homeostasis, and psychological wellness 5. Reduced testosterone levels or hypogonadism results in sexual dysfunction that includes erectile dysfunction (ED), premature ejaculation (PE), and decreased interest or lack of desire are the most common sexual dysfunctions 6, 7. Globally, 322 million men are predicted to have ED by 2025, an increase from 152 million in 1995 8. PE affects 30% to 50% of males 9. ED and PE are intricately related to each other with a bidirectional relationship. ED has been reported as the major risk factor for about 36% to 50% of incidences of PE 10.

Numerous Indian medicinal herbs have been used in traditional medicine to treat sexual disorders and improve men's quality of life, sperm count and motility, and sexual performance 11, 12. A proprietary blend of *Punica granatum* fruit rind and the seeds of *Theobroma cacao*, LN18178, synergistically increased steroidogenesis in mouse MA-10 Leydig cells and decreased aromatase enzyme activity in JEG-3 human choriocarcinoma cells. Furthermore, in a proof-of-concept preclinical study, LN18178 supplementation increased serum testosterone, luteinizing hormone levels, and semen quality (volume, sperm count, and motility) in young adult male Sprague Dawley rats (unpublished observation). Earlier, a clinical study in young male volunteers (age 21-35 yrs.) demonstrated that LN18178 supplementation increased testosterone (total and free) levels, and the volunteers enhanced their muscle mass and strength 13. Next, in another independent human trial, LN18178 also significantly increased serum testosterone (total and free) levels and ameliorated the aging male symptoms (AMS) that suggested improvements in psychological, physical, and sexual behavior and activities in the participants (age: 36-55 yrs.) 14.

Pomegranates (*Punica granatum* L.) are associated with fertility, regeneration, and endurance of life 15. Pomegranate is a rich source of active antioxidant phytochemical compounds like ellagic acid, gallic acid, quercetin, myricetin, and flavonoids such as anthocyanidins, cyanidins, luteolin, and pelargonidins, etc. 16. Pomegranate juice has been reported to increase sperm count, motility, and viability in vivo 17. Furthermore, pomegranate juice supplementation increased sperm count and mobility in the epididymis and reduced poor-quality sperm in male rats. These beneficial effects include increased intra-cavernosal blood flow, smooth muscle relaxation, and erectile activity against oxidative stress 18. Earlier, a clinical study demonstrated improved erectile function in pomegranate juice-supplemented individuals compared to placebo 19.

*Theobroma cacao* or cocoa seeds are rich in phenolic antioxidant flavonoids like catechins, epicatechins, procyanidin B1 and -B2, quercetin, luteolin, vitexin, phenolic acids, etc. 20. Epicatechins are primarily responsible for their beneficial impact on the vascular endothelium by upregulating nitric oxide (NO) production. *T. cacao* improves insulin sensitization. Additionally, *T. cacao* stimulates changes in redox-sensitive signaling pathways and the immune response. It also benefits nerve injury, skin protection from ultraviolet (UV) radiation, satiety, cognitive function, and mood elevation 21. A diet containing *T. cacao* seeds significantly improved the semen quality in rabbit bucks 22.

The objective of the present study was to explore whether LN18178 supplementation improves sexual function in aging male volunteers. We conducted an 84-day randomized, double-blind, placebo-controlled clinical study that demonstrated enhanced sexual and erectile functions in LN18178-supplemented aging male participants with low sexual desire and mild to moderate levels of erection difficulty.

## Materials and methods

### A proprietary phytoceutical composition, LN18178 (TesNor^®^)

LN18178, a patented (PCT/IN2019/050361) synergistic combination of *Punica granatum* fruit rind and *Theobroma cacao* seed extracts (4:1 w/w) (batch # N22050248, mfg. on May 2022), was manufactured at a good manufacturing practice (cGMP)-certified facility of Laila Nutraceuticals, Vijayawada, India. Taxonomically authenticated voucher specimens of *Punica granatum* fruit rind (LNH6341) and *Theobroma cacao* seeds (LNH6924) are archived in the Taxonomy Department, Laila Nutraceuticals (Vijayawada, India). The extract blend was formulated to a free-flowing powder using 25% excipients (w/w) and standardized to a minimum of 3.5% punicalagins and 0.5% theobromine, affirmed using high-performance liquid chromatography (HPLC). Detailed descriptions of the raw materials, collection, extraction procedures, standardization, and phytochemical analysis of the plant were provided earlier 13.

### Ethical conduct

**Figure 1:**
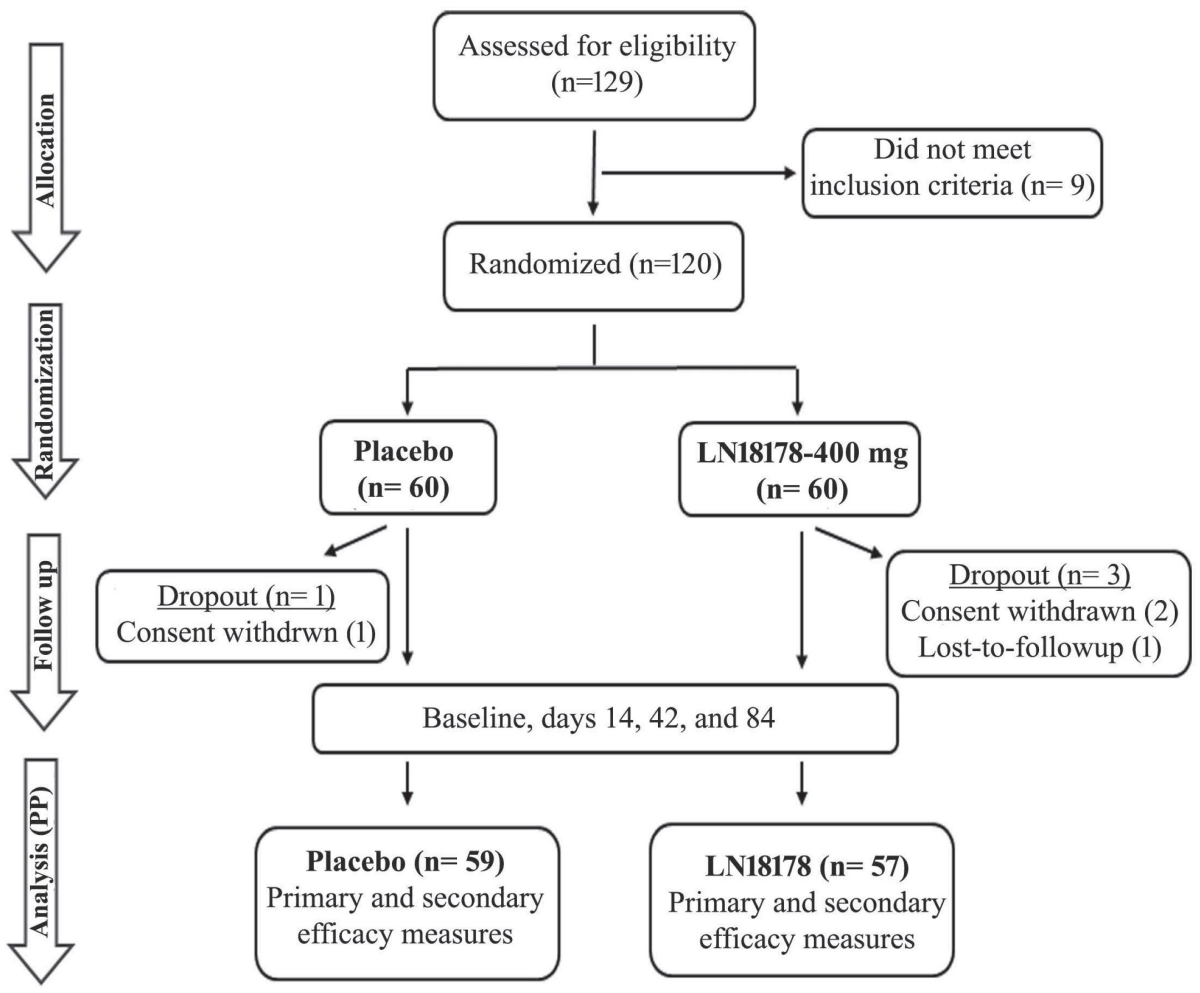
A CONSORT diagram shows the flow of the study. The primary and secondary efficacy measures are described in the materials and methods. The safety evaluations, which included complete serum biochemistry, hematology, urine analysis, and vitals, were performed at the screening and the end of the study (day 84).

This double-blind, placebo-controlled clinical trial was registered with the Clinical Trial Registry of India (Registration No: CTRI/2023/03/050315; March 03, 2023) and approved by the institutional ethics committee (IEC) (ECR/1611/Inst/UP/2021) of Kashi Medicare and Upendra Medicare (Varanasi, Uttar Pradesh, India) on January 28, 2023. The study strictly followed the ethical principles of the Declaration of Helsinki, International Conference on Harmonization (ICH) - Good Clinical Practice (GCP) guidelines. The study flow is presented following the recommendations of Consolidated Standards of Reporting Trials (CONSORT) (Figure 1).

### Participant enrollment, consent, randomization, and blinding

Healthy and recreationally active aging men (age: 40-70 years; BMI: 20-29 kg/m^2^; serum total testosterone levels: ≥ 300 ng/dL) with mild erectile dysfunction (IIEF scores between 17 and 25) and low sexual desire, as assessed using self-reported International Index of Erectile Function (IIEF) questionnaire. The participants were in monogamous sexual relationships, and all of them complied with the inclusion-exclusion criteria of the study (Supplementary Table S1).

Each participant read the subject information sheet detailing the study procedures, aims, methodology, potential risks, and anticipated benefits. Then, all participants signed the IEC-approved informed consent form.

The enrolled participants (n=120) were equally allocated to either placebo or LN18178 groups using permuted block-randomization codes generated by the PROC-PLAN procedure in the Statistical Analysis System (SAS) program. The randomization details were controlled by an independent authorized designatory; the investigators, study monitors, and study participants were blinded to the randomization and treatments. Randomization codes were broken only after locking the data following the completion of the study.

### Sample size and Power calculation

The sample size was calculated using Stata Statistical Software (StataCorp LLC. 2019, College Station, TX). Sixty subjects per treatment group were assumed to provide 90% power to detect a treatment effect at the end of the study (day 84) Rmax(µm) change from baseline at a one-sided significance level of 0.05%. Based on an earlier study 23, assuming a pooled standard deviation of 1.31 to achieve the power of 90% and at 95% CI for detecting a difference in means between two groups of 0.96, the total minimum sample size was estimated to be 110 (55 in each group, two groups). This study recruited 120 participants (60 per group) with an assumption of a 10% dropout during the study.

### Placebo or LN18178 Supplementation

The coded placebo or LN18178 capsules supplied to all recruited subjects contained either the investigational products (IP), LN18178, or matched placebo capsules of identical sizes, weights, and colors. Recruited subjects were advised to orally consume one placebo or LN18178 capsule (400 mg/day) with breakfast for 84 consecutive days. Each placebo capsule contained (w/w) brown dextrin (50%) and maltodextrin (50%).

### Follow-up visits

Following screening and enrollment (visit 1), the enrolled subjects visited the site for baseline evaluations (visit 2). This study had three follow-up evaluations on days 14 (visit 3), 42 (visit 4), and 84 (visit 5) of treatment.

### Compliance

The placebo and LN18178 capsules were stored at room temperature in a dry, cool, and dark place. The project coordinators exclusively distributed the placebo and treatment capsules to the recruited volunteers at baseline and on days 14 and 42 of the study. They maintained the data entry and were endorsed regularly by the principal investigator (PI). The PI regularly signed the accountability log. Study participants were advised to keep their routine regular diets and refrain from consuming any vitamins or beverages that were claimed to be ergogenic and enhance sexual function. All subjects regularly maintained individual daily diaries and recorded details of food and capsule intake, daily activities, and any or all adverse or untoward events. These daily diaries were routinely checked by the project coordinators and endorsed by the PI.

The project coordinators and PI periodically counseled the study participants to ascertain the maximum possible adherence to the study protocol. The participants returned all unused capsules at each follow-up visit, and their attendance at each visit was recorded to ensure the participants' IP-related and participant compliance with the study protocol.

The PI determined the physical health of all study participants by checking for signs of any adverse drug reaction. Safety was ascertained by medical checkups and laboratory evaluations at baseline and each follow-up visit.

### Subject withdrawal criteria

The withdrawal of subjects from the study was considered if the subject had withdrawn consent or the investigator considered withdrawal in case of non-compliance with IP or protocol violation or loss to follow-up. The withdrawal was also considered in the subjects' interest due to tolerability issues, including serious adverse events. The reasons for withdrawal or dropout from the study were recorded in the case report form.

### Concomitant medication

All subjects routinely maintained the intake records of all concomitant medications, including prescription, non-prescription, and over the counter (OTC) medications, and the study coordinators recorded these details on the case report forms (CRFs).

### Efficacy measurements

#### Derogatis Interview for Sexual Functioning-Self Reported-Male (DISF-SR-M)

The scores of the DISF-SR-M questionnaire 24 were the primary efficacy outcome measure that evaluated the improvements in sexual functions of the participants on days 14, 42, and 84 of LN18178 supplementation compared to the baseline. The 25-question DISF-SR-M questionnaire is divided into five domains: sexual cognition/fantasy, sexual arousal, sexual behavior/experience, orgasm, and sexual drive/relationship. The sexual cognition/fantasy, sexual arousal, sexual behavior/experience domain questions, and two sexual drive/relationship-related questions are scored on a 9-point scale between 0 and 8. The orgasm-related questions and two questions from the sexual drive/relationship domain are scored between 0 (not at all) and 4 (extreme).

#### The International Index of Erectile Function (IIEF)

The IIEF questionnaire is a self-reported 15-item instrument to assess male sexual function. The IIEF consists of five domains: erectile function, orgasmic function, sexual desire, intercourse pleasure, and overall satisfaction. IIEF score evaluation is the gold standard for clinical efficacy assessment in erectile and sexual function studies 25, 26.

#### Erection hardness score (EHS)

EHS is a valid and reliable tool for scoring erection hardness 27. This single-item self-reported score classifies the severity of erectile dysfunction (ED) into four grades:

(a) Grade 1 represents no enlargement and lack of hardness upon sexual stimulation.

(b) Grade 2 indicates that it is not hard enough to penetrate.

(c) Grade 3 indicates sufficient for penetration but not completely hard.

(d) Grade 4 indicates normal erection in hardness and rigidity.

#### The Multidimensional Fatigue Inventory (MFI)

The MFI is a 20-item self-reported instrument designed procedure to assess fatigue. It measures general fatigue, physical fatigue, mental fatigue, reduced motivation, and reduced activity 28. The participants scored based on their perception of fatigue on a scale of 1 to 5, the higher the score, the higher the level of fatigue. MFI scores were evaluated at baseline and on days 42 and 84 of the study.

#### The Pittsburgh Sleep Quality Index (PSQI)

The participants were assessed for their sleep quality at baseline and on days 42 and 84 of treatment using a self-rated Pittsburgh Sleep Quality Index (PSQI) questionnaire. The PSQI reliably assesses sleep quality and disturbances in clinical practice and research setups. The PSQI measures sleep quality, latency, duration, habitual sleep efficiency, sleep disturbances, use of sleep medication, and daytime dysfunction 29. Each item is scored between 0 and 3; the combined score of seven domains presents the global score. A global score greater than five indicates poor sleep quality.

#### General Health Survey (GHS)

The participants' feedback on their libido, muscular mass, muscle strength, energy, stamina, and sleep were captured on a 10-cm scale of a general health survey (GHS) questionnaire. A score of 1 indicates "not satisfied," and a 10 indicates "extremely satisfied.”

#### Hand-grip strength

The hand-grip strength of the dominant hand of the participants was measured using a digital dynamometer (INCO Instruments & Medical Devices Pvt. Limited, Ambala, India). The measurements were taken in the sitting position with the forearm extended on a table and bent at 90° with the elbow. The participants squeezed their grips as hard as possible without any jerking motion; the best measurement was recorded among three performances at 2-minute intervals.

#### Six-minute walk test (6MWT)

6MWT was performed to measure the submaximal level of functional activity of the participants following the American Thoracic Society (ATS) guidelines 30. The participants walked on a 25-meter length of a well-ventilated and flat surface as quickly as possible for six minutes; the walked distance was recorded as a six-minute walk distance (6MWD). They were allowed for rest periods, but the time was included in the test duration. The participants were verbally encouraged during the test. They were allowed to withdraw from the test in case of any discomfort, including chest pain, extreme shortness of breath, or leg cramps.

The IIEF, EHS, 6MWT, GHS, and hand-grip strength evaluations were performed at baseline and on each follow-up visit of the study.

### Safety assessments

Total blood chemistry was carried out during screening and at the end of the study, including an array of hematological, serum biochemical, and urinary analyses. Urine and blood chemistry analyses were conducted using the VITROS^®^ 5600 integrated system (Dry Chemistry analyzer, Ortho Clinical Diagnostics, Linden, NJ, USA). Fasting blood glucose, serum creatinine, uric acid, creatine kinase (CK), blood urea nitrogen (BUN), serum bilirubin, aspartate aminotransferase (AST), alanine transaminase (ALT), alkaline phosphatase (ALP), sodium, potassium, and serum albumin were the clinical chemistry parameters. The hematological parameters included hemoglobin, platelet count, total leukocyte count (TLC), red blood cells (RBC), erythrocyte sedimentation rate (ESR), and differential count. Color, specific gravity, pH, glucose, protein, and RBC were evaluated in urinalysis. Microscopic examinations were conducted using a light microscope (Olympus Opto Systems India Pvt. Ltd., New Delhi, India). At each visit, the participants' vital signs, blood pressure (systolic and diastolic), pulse rate, respiration rate, and oral temperature were recorded.

### Statistical analysis

The data are presented as mean ± SD. The per-protocol (PP) analysis was performed using the data of the subjects who completed the study: placebo (n=59) and LN18178 (n=57). Intragroup comparisons were analyzed using paired t-test, and Wilcoxon signed rank test for normal and nonnormal data, respectively. Intergroup comparisons were performed using Analysis of Covariance (ANCOVA) and rank ANCOVA for normal and non-normal data respectively. ANCOVA was used to adjust the baseline differences between the groups. For non-normal data, rANCOVA was performed for covariate adjustments and to reject the type 1 error (false positives). For safety analysis, paired t-tests and independent t-tests were used for intragroup and intergroup analysis. All hypotheses were tested at a significance level of 0.05 and 95% confidence interval (CI). The effect size (Cohen's d) was calculated as the product of the mean difference between the groups to pooled standard deviation (pooled SD).

## Results

One hundred and twenty aging males (age: 52.95 ± 8.52 Y; BMI: 24.71 ± 1.32 kg/m^2^; Race: Asian) were enrolled in the study, and they were equally allocated to the placebo and LN18178 groups; each group consisted of 60 participants. None of them had a smoking or tobacco consumption history. The comparative analyses of the baseline demographic parameters (age, height, body weight, BMI, serum total testosterone) of the groups suggest that there was no statistical difference between the groups (Table 1). Overall, four participants dropped out from the study; one from the placebo group and two from the LN18178 group withdrew their consents after recruitment, and one subject in the LN18178 group did not report after the baseline visit. The per protocol (PP) analyses are presented using the placebo (n= 59) and LN18178 (n= 57) for the efficacy evaluation of supplementation.

### DISF-SR-M scores

Table 2 summarizes gradual increases of the DISF-SR-M total and domain scores in the LN18178-supplemented volunteers from day 14 through the end of the study. Although the improvements in the placebo are significant (vs. baseline), the between-the-group comparison analysis reveals that LN18178 supplementation significantly (vs. placebo) increased the domain and total DISF-SR-M scores in the participants. Post-trial, the LN18178 and placebo groups showed 121.93% and 42.42% increases in DISF-SR scores, respectively, from baseline. The improvement in LN18178 (vs. placebo) is significant (P < 0.0001; Cohen'd: 0.88, 2.06, and 4.99 on days 14, 42, and 84, respectively) (Table 2). Interestingly, the comparisons (placebo vs. LN18178) between the improvements (from baseline) in scores of two specific questions on morning erection (Q 2.1) and frequency of sexual activity (Q 3.5) are significant (P < 0.001: Cohen'd: 2.77 and 3.03 respectively for Q 2.1 and 3.5, respectively) starting from day 14 till the end of the study (Table 2). In men, morning erection is negatively associated with physical and psychological stress 31, and the frequency of sexual activity determines the sexual relationship with a partner, general well-being, and health 32.

### IIEF scores

Table 3 demonstrates gradual and significant improvements in all domains (erectile function, orgasmic function, sexual desire, intercourse satisfaction, and overall satisfaction) and total IIEF scores in the LN18178-supplemented subjects. LN18178 supplementation increased the total IIEF scores by 21.25%, 42.30%, and 72.02%. In contrast, compared to baseline, the placebo group showed 6.79%, 18.80%, and 35.97% increases on days 14, 42, and 84, respectively. These changes are significant (P < 0.0001) in the within-the-group (vs. baseline) and between-the-group (vs. placebo) comparison analyses (Table 3). The Cohen'd values for improvement in IIEF total scores are 0.15, 2.52, and 3.98, respectively. The comparison analysis between the changes (from baseline) in the groups showed significant improvements in total and the IIEF domain scores in the LN18178-supplemented participants on the follow-up visits of the study (Table 3).

### EHS scores

Similarly, on days 14, 42, and 84 of LN18178 supplementation, the erection hardness (EHS) scores were significantly (P < 0.0001) increased by 22.28%, 54.46%, and 80.69% from baseline, whereas the placebo group showed only 0.51%, 5.58%, and 7.61% increases, respectively. The changes in the placebo group are not significant (vs. baseline). Between-the-group comparison analysis on the net scores and the changes from baseline reveal that the improvements in the LN18178 group on the follow-up visits are significant (P < 0.0001, vs. placebo) (Table 3). The Cohen's d values for improvement in EHS scores are 0.78, 1.59, and 2.51, respectively,

### MFI scores

Data analysis of multi-dimensional fatigue inventory (MFI) scores revealed that post-trial LN18178 significantly decreased the total MFI and selected domain scores (general fatigue, physical fatigue, and mental fatigue) as compared to baseline (Table 4). No significant change in total MFI scores was recorded in the placebo group following 84 days of supplementation. Relative to placebo, the MFI total scores were significantly decreased on days 42 (Cohen's d -0.26) and 84 (Cohen's d -0.62). However, post-trial, the MFI total score and its improvement (change from baseline) in the LN18178-supplemented group was not statistically significant (vs. placebo) (Table 4).

### PSQI scores

Similarly, the PSQI global score in the LN18178-supplemented group was significantly (P < 0.05) reduced on days 42 and at the end of the study as compared to baseline and placebo (Table 5). Post-trial, the domain scores, such as subjective sleep quality, sleep disturbances, and daytime dysfunction in the LN18178-supplemented group, were significantly improved (p<0.05) when compared directly to baseline and placebo (Table 5). Also, post-trial, analysis showed significant improvements (change from baseline) in the global score, and these domain scores in the LN18178 group (vs. placebo) were substantial. Compared to placebo, the Cohen's 'd' values for improving sleep quality (Global PSQI scores) were 0.65 and 1.08, respectively (Table 5).

### Hand-grip strength

**Figure 2:**
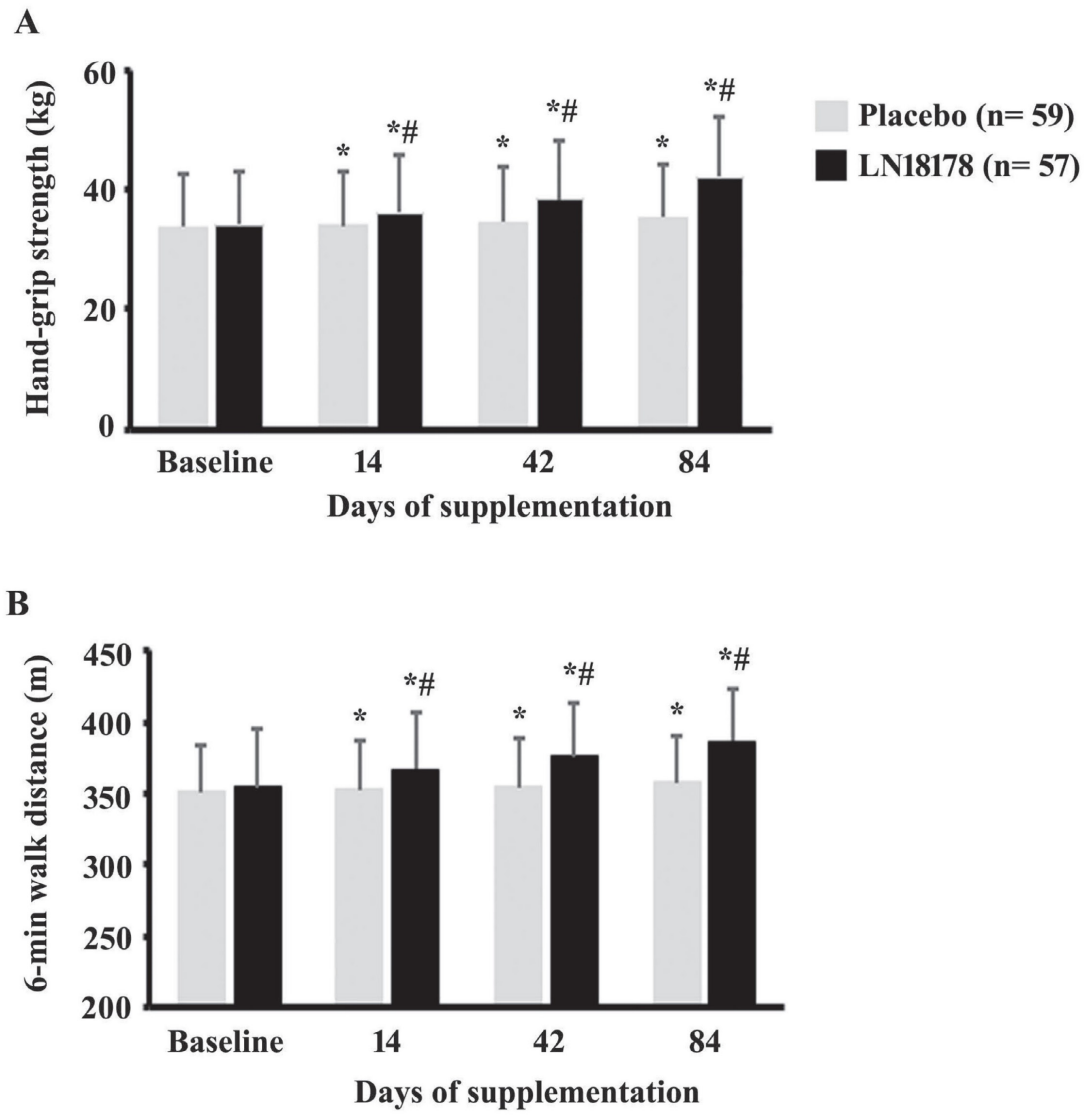
The bar diagrams present mean ± SD of the (A) hand-grip strength and (B) six-minute walk distance (m) in the placebo (n=59) and LN18178 (n=57) groups at baseline and on 14, 42 and 84 days of the study. * and # indicate significance (P < 0.05) in 'within the group' (vs. baseline) and 'between the group' (vs. placebo) comparison analyses using paired t-test and ANCOVA, respectively.

On days 14, 42, and 84 of the investigation, the hand-grip strength in the LN18178 group were increased by 5.98% (P < 0.0001), 12.72% (P < 0.0001), and 23.83% (P < 0.0001), in comparison, the increases in the placebo group were 0.77% (P = 0.0029), 2.70% (P < 0.0001), and 5.01% (P < 0.0001), respectively, as compared to baseline (Figure 2A). These improvements in the LN18178-supplemented volunteers are significant (P < 0.0001) when compared with the placebo on days 14, 42, and 84 of the study (Figure 2A).

### Six-minute walk test

LN18178-supplemented participants significantly (P < 0.0001) improved (P < 0.0001) the absolute walked distance in the six-minute walk test (SMWT) (Figure 2B). From baseline, the increases in the ADW by the LN18178 subjects were 3.56%, 5.93%, and 9.06% on days 14, 42, and 84 of supplementation. These improvements were also significant (P < 0.0001) compared to the placebo. In placebo, on days 14, 42 and 84, the improvements were 0.44% (P = 0.0003), 0.99% (P < 0.0001, and 1.97% (P < 0.0001), respectively, from baseline (Figure 2B).

### GHS scores

Comparative analysis of general healthy survey (GHS) scores revealed that the GHS total and the domain scores were significantly (P < 0.0001) increased in the LN18178 group on days 14, 42, and 84 of supplementation as compared to the baseline and placebo (Supplementary Table S2).

### Adverse events and concomitant medication

During the intervention, in the placebo, two subjects (one each) reported bloating and abdominal pain), and in the LN18178 group, three subjects (one each) reported an incidence of vomiting, nausea, or headache. However, these events were minor and transient (Supplementary Table S3). No participant reported any concomitant medication usage during the study.

### Safety assessments

At the screening visit the participants' total serum biochemistry, hematology, urine analysis, and vital parameters were within the normal ranges. At the end of the study, these safety parameters remained within the normal ranges (supplementary Table S4).

## Discussion

The major outcome of the present study indicates that LN18178 supplementation is safe, and it increased overall sexual function (DISF-SR-M total score) and associated behavioral functions, including sexual cognition, arousal, sexual behavior, orgasm, and desire (Table 2). The daily consumption of LN18178 over 84 consecutive days is safe and well-tolerable by the participants. None of the study participants reported any major adverse events; their complete blood biochemistry, including liver, cardiovascular, and kidney functions, lipid profiles, hematology, and urinalysis parameters, were within the normal ranges. These safety observations were consistent with the earlier clinical studies 13, 14. Importantly, LN18178 has shown comprehensive safety, as affirmed by a ninety-day sub-chronic oral toxicity study in rats and *in vitro* and *in vivo* genetic toxicology studies 33. LN18178 is a food-derived ingredient; *P granatum* fruit rind powder and extracts are used in the dairy industry 34, and *T. cacao* beans are widely used in confectionaries 35.

Earlier clinical investigations conferred that LN18178, a synergistic phytoceutical composition, significantly increased serum total and free testosterone levels in young and aging male volunteers. LN18178 supplementation also enhanced the participants' muscle mass and strength [13. 14]. Cell-based *in vitro* studies demonstrated that this phytoceutical composition increased testosterone production via enhancing steroidogenesis in MA-10 mouse Leydig cells by upregulating Steroid acute regulatory protein (StAR) and cytochrome P450 family 17 subfamily A member 1 (CYP17A1) and reducing the conversion of testosterone to estradiol by inhibiting aromatase activity (unpublished data).

Testosterone is the integral androgenic and anabolic endocrine factor for men that regulates the development of male sexual traits and maintains and enhances sexual function and body composition 36. In adult males, sexual function and performance are well-coordinated and regulated by physiological and psychological or emotional factors. Although sexual function is a multi-factorial process, testosterone plays a major and central role in modulating the emotional and neuro-physiological control of sexual arousal, erection and penetration, ejaculation, satisfaction, and overall performance 37. Low testosterone levels are strongly associated with reduced sexual function, including impaired erectile function, libido, and desire in men. Testosterone replacement therapy (TRT) in hypogonadal men has been shown as a therapeutic strategy for the clinical management 37, 38. However, due to serious side effects, including a rise in blood pressure, liver toxicity, increased risk of heart attack, and stroke, the US FDA recommendation limits the TRT or androgenic-anabolic steroids (AAS) therapy for selected medical conditions rather than age-related androgen deficiency 39. In this context, a gradual rise in public attention to natural, plant-based diets and therapies for improving male sexual function, including increased hormone levels, erectile function, and libido, is worth mentioning 40, 41.

LN18178 supplementation has improved multi-dimensional fatigue inventory (MFI) and Pittsburgh Sleep Quality Index (PSQI) scores, indicative of reduced fatigue/stress and improved sleep quality. These improvements indicate the psychological benefits of LN18178 in the participants. Poor psychological status and low testosterone significantly contribute to impaired erectile function pathologies 42. Interestingly, the increased frequency of morning erection and reduced physical and mental fatigue domain scores of the MFI questionnaire suggest reduced physical and psychological stress in the LN18178-supplemented participants. Morning erections in men are rapid eye movement (REM)-sleep-related normal physiological phenomena that are coordinated primarily by testosterone levels and have been negatively associated with physical and mental stress 43. LN18178 improved the overall erectile function of the participants. Sustainable penile erection is a muscular response in coordination with the central and peripheral neural control involving endocrine factors like testosterone 44, 45. Testosterone plays a pivotal role in regulating male sexual performances via central and autonomic responses 37 and indirectly increases penile blood flow and improves erection by reducing alpha-adrenergic activity in the vascular smooth muscles of the corpus cavernosum 44, 45.

Other important observations from this study are that LN18178 substantially increased the six-minute walk distance and hand-grip strength (HGS), suggesting an enhanced aerobic capacity and endurance 46 and increased muscle strength 47 in the participants. Improvements in the 6MWT indicate increased cardiopulmonary function with improved aerobic capacity and endurance 46, 48. Improvements in the isometric muscular strength (as HGS measurement) in the LN18178-supplemented participants corroborate earlier observations 13, 14. HGS determines aging-related muscle loss and measures neuromotor function and overall physical fitness in an aging population 49. Testosterone is an anabolic hormone; a lower testosterone level is associated with muscle weakness in aging men 47. Testosterone increases mitochondrial function in muscles via enhanced mitochondrial gene expression, thus helping improve energy metabolism in the muscles and preventing the gradual loss of skeletal muscle mass in aging. Elevated levels of mitochondrial function and prevention from muscle loss help improve muscle strength, endurance, and recovery 50. Testosterone also plays a pivotal role in balancing multi-dimensional psychological networks of mood, behavior, self-perception, and perceived quality of life in men across the age range [51. Overall, the present and earlier observations 13, 14 on increased muscle strength and endurance affirm an anabolic effect of LN18178; also, the combined observations on increased HGS and GHS scores and global PSQI scores suggest a possible role of LN18178 in increasing vitality, vigor, and well-being in the participants.

The present study has a few limitations. This study did not measure the semen parameters, such as semen volume, sperm count, motility, etc. A future investigation on young male volunteers would be interesting. The present study did not test the efficacy of LN18178 supplementation on participants' body composition. Testosterone regulates metabolic function, age-related muscle and bone loss, and fat accumulation 52. However, based on the present and earlier observations on the increased testosterone levels in the participants, we anticipate that LN18178 would improve body composition, thus warranting a more extended duration investigation.

## Conclusion

LN18178 (TesNor^®^) is a safe and well-tolerated phytoceutical composition containing a combination of *Punica granatum* fruit rind and *Theobroma cacao* seed extracts. The present randomized, double-blind, placebo-controlled study data affirm that LN18178 consumption increases sexual function, erectile function, and libido and improves psychological well-being, muscle strength, neuromotor function, and general well-being in aging males. This botanical supplementation holds a potential promise to be an effective strategy in clinical practice to improve male sexual function and physical and psychological health in aging adults. Further research is warranted to evaluate the efficacy of LN18178 in male fertility.

## Supplementary Material

Supplementary tables.

## Figures and Tables

**Table 1:** Baseline and demographic characteristics

		Mean ± SD	P value (vs. placebo)	95% CI (vs. placebo)
Age (years)				
Placebo		51.88 ± 8.28	-	
LN18178		54.02 ± 8.69	0.1710	-0.93, 5.21
Race (Gender)				
Placebo		60 Asian (male)	-	-
LN18178		60 Asian (male)	-	-
Height (cm)				
Placebo		163.42 ± 4.06	-	
LN18178		163.72 ± 5.36	0.7304	-1.42, 2.02
Weight (Kg)				
Placebo		66.20 ± 4.61	-	
LN18178		66.02 ± 4.77	0.8353	-1.52, 1.88
BMI (kg/m^2^)				
Placebo		24.78 ± 1.38	-	
LN18178		24.63 ± 1.27	0.5210	-0.33, 0.63
Total Testosterone (ng/dL)				
Placebo		418.52 ± 40.76	-	
LN18178		433.40 ± 43.56	0.0557	-0.37, 30.14
Fasting blood glucose (mg/dL)				
Placebo	87.65 ± 6.16		-	-
LN18178	87.60 ± 7.08		0.9672	-2.35, 2.45
Smoking history (past/present)				
Placebo		0	-	-
LN18178		0	-	-
				

Values present mean ± standard deviation (SD); placebo (n=60) and LN18178 (n=60). CI: Confidence interval. A P value < 0.05 (independent t-test) was considered significant.

**Table 2:** Assessment of DISF-SR-M scores

	Evaluation	Mean ± SD	P value (vs. baseline)	P value (vs. placebo)	95% CI vs. Baseline	95% CI vs. Placebo	P value, change from baseline (vs. placebo)
*Sexual cognition*							
Placebo	Baseline	11.64 ± 2.77	-	-	-	-	-
	Day 14	12.69 ± 2.45	< 0.0001	-	0.10, 2.00	-	-
	Day 42	12.37 ± 2.37	0.0287	-	-0.21, 1.67	-	-
	Day 84	14.41 ± 3.92	< 0.0001	-	1.53, 4.01	-	-
LN18178	Baseline	12.07 ± 2.08	-	0.4120	-	-0.47, 1.33	-
	Day 14	15.04 ± 3.01	< 0.0001	< 0.0001	2.01, 3.93	1.34, 3.36	< 0.0001
	Day 42	17.88 ± 2.54	< 0.0001	< 0.0001	4.95, 6.67	4.61, 6.41	< 0.0001
	Day 84	26.21 ± 2.79	< 0.0001	< 0.0001	13.23, 15.05	10.54, 13.06	< 0.0001
*Sexual arousal*							
Placebo	Baseline	10.98 ± 3.03	-	-	-	-	-
	Day 14	12.05 ± 2.35	< 0.0001	-	0.08, 2.06	-	-
	Day 42	15.08 ± 2.52	< 0.0001	-	3.08, 5.12	-	-
	Day 84	15.20 ± 2.5	< 0.0001	-	3.21, 5.23	-	-
LN18178	Baseline	10.91 ± 3.01	-	0.8999	-	-1.04, 1.18	-
	Day 14	14.58 ± 3.01	< 0.0001	< 0.0001	2.55, 4.79	1.54, 3.52	< 0.0001
	Day 42	18.39 ± 2.88	< 0.0001	< 0.0001	6.39, 8.57	2.32, 4.30	< 0.0001
	Day 84	26.54 ± 2.8	< 0.0001	< 0.0001	14.55, 16.71	10.36, 12.32	< 0.0001
*Sexual behaviour*							
Placebo	Baseline	10.54 ± 2.36	-	-	-	-	-
	Day 14	12.02 ± 1.94	< 0.0001	-	0.69, 2.27	-	
	Day 42	15.17 ± 2.21	< 0.0001	-	3.80, 5.46	-	-
	Day 84	15.08 ± 1.99	< 0.0001	-	3.74, 5.34	-	-
LN18178	Baseline	10.12 ± 2.88	-	0.3916	-	-0.55, 1.39	-
	Day 14	13.53 ± 2.67	< 0.0001	< 0.0001	2.38, 4.44	0.65, 2.37	< 0.0001
	Day 42	17.54 ± 2.24	< 0.0001	< 0.0001	6.46, 8.38	1.55, 3.19	< 0.0001
	Day 84	26.12 ± 2.54	< 0.0001	< 0.0001	14.99, 17.01	10.20, 11.88	< 0.0001
*Orgasm*							
Placebo	Baseline	11.32 ± 2.87	-		-	-	-
	Day 14	12.64 ± 3.23	< 0.0001		0.21, 2.43	-	
	Day 42	13.75 ± 3.06	< 0.0001	-	1.35, 3.51	-	-
	Day 84	17.39 ± 2.29	< 0.0001	-	5.12, 7.02	-	-
LN18178	Baseline	11.56 ± 3.05		0.6644	-	-0.85, 1.33	-
	Day 14	13.81 ± 2.46	<0.0001	<0.0001	1.22, 3.28	-0.60, 1.52	0.0018
	Day 42	15.16 ± 2.22	<0.0001	0.0018	2.61, 4.59	0.42, 2.40	0.0195
	Day 84	20.88 ± 2.01	<0.0001	<0.0001	8.36, 10.28		<0.0001
*Sexual drive*							
Placebo	Baseline	8.97 ± 1.61	-	-	-	-	-
	Day 14	10.34 ± 1.94	<0.0001	-	0.72, 2.02	-	-
	Day 42	11.05 ± 1.98	<0.0001	-	1.42, 2.74	-	-
	Day 84	14.05 ± 2.37	<0.0001	-	4.34, 5.82	-	-
LN18178	Baseline	8.49 ± 1.91		0.1496	-	-0.17, 1.13	-
	Day 14	10.95 ± 1.85	<0.0001	0.00411	1.76, 3.16	-0.09, 1.31	0.0004
	Day 42	14.56 ± 2.08	<0.0001	<0.0001	5.33, 6.81	2.76, 4.26	<0.0001
	Day 84	18.23 ± 1.6	<0.0001	<0.0001	9.09, 10.39	3.43, 4.93	<0.0001
*Total score*							
Placebo	Baseline	53.46 ± 9.25	-	-	-	-	-
	Day 14	59.75 ± 8.18	<0.0001	-	4.81, 10.53	-	-
	Day 42	67.42 ± 7.47	<0.0001	-	10.89, 17.03	-	-
	Day 84	76.14 ± 7.22	<0.0001	-	13.58, 19.20	-	-
LN18178	Baseline	53.16 ± 10.34		0.8694	-	-3.31, 3.91	-
	Day 14	67.89 ± 10.37	<0.0001	<0.0001	10.89, 18.57	4.71, 11.57	<0.0001
	Day 42	83.53 ± 8.16	<0.0001	<0.0001	26.91, 33.83	13.23, 18.99	<0.0001
	Day 84	117.98 ± 9.56	<0.0001	<0.0001	61.12, 68.52	38.73, 44.95	<0.0001
*Morning erection (Q 2.1)*							
Placebo	Baseline	2.34 ± 0.92	-	-	-	-	-
	Day 14	2.53 ± 0.73	0.0245	-	-0.11, 0.49	-	-
	Day 42	3.02 ± 0.75	< 0.0001	-	0.37, 0.99	-	-
	Day 84	2.95 ± 0.86	< 0.0001	-	0.29, 0.93	-	-
LN18178	Baseline	2.19 ± 0.95	-	-	-	-0.19, 0.49	-
	Day 14	3.04 ± 0.84	< 0.0001	< 0.0001	0.52, 1.18	0.22, 0.80	< 0.0001
	Day 42	3.58 ± 1.02	< 0.0001	0.0004	1.02, 1.76	0.23, 0.89	0.0009
	Day 84	5.26 ± 0.81	< 0.0001	< 0.0001	2.74, 3.40	2.00, 2.62	< 0.0001
*Sexual activity (Q 3.5)*							
Placebo	Baseline	2.22 ± 0.91	-	-	-	-	-
	Day 14	2.63 ± 0.72	< 0.0001	-	0.11, 0.71	-	-
	Day 42	3.22 ± 0.85	< 0.0001	-	0.68, 1.32	-	-
	Day 84	3.22 ± 0.67	< 0.0001	-	0.71, 1.29	-	-
LN18178	Baseline	2.28 ± 0.96	-	-	-	-0.28, 0.40	-
	Day 14	2.95 ± 0.88	< 0.0001	0.0186	0.33, 1.01	0.03, 0.61	0.0738
	Day 42	3.88 ± 0.87	< 0.0001	< 0.0001	1.26, 1.94	0.34, 0.98	0.0097
	Day 84	5.37 ± 0.75	< 0.0001	< 0.0001	2.77, 3.41	1.89, 2.41	< 0.0001

Values present mean ± SD. placebo (n=59) and LN18178 (n=57). CI: Confidence interval; P < 0.05 was considered as statistically significant for 'within the group' and 'between the groups' comparison analysis using paired t test and ANCOVA, respectively, as described in materials and methods.

**Table 3:** Assessment of International Index of Erectile Function scores (IIEF) & Erection Hardness Scores (EHS)

	Evaluation	Mean ± SD	P value (vs. baseline)	P value (vs. placebo)	95% CI (vs. Baseline)	95% CI (vs. Placebo)	P value, change from baseline (vs. placebo)
*IIEF- Erectile function*							
Placebo	Baseline	17.80 ± 0.98	-	-	-	-	-
	Day 14	18.22 ± 1.47	0.0722	-	-0.04, 0.88	-	-
	Day 42	18.78 ± 1.47	< 0.0001	-	0.52, 1.44	-	-
	Day 84	20.44 ± 1.67	< 0.0001	-	2.14, 3.14	-	-
LN18178	Baseline	17.81 ± 0.81		0.9341	-	-0.32, 0.34	-
	Day 14	20.07 ± 1.49	< 0.0001	< 0.0001	1.81, 2.71	1.31, 2.39	< 0.0001
	Day 42	22.28 ± 1.71	< 0.0001	< 0.0001	3.97, 4.97	2.91, 4.09	< 0.0001
	Day 84	25.54 ± 1.73	< 0.0001	< 0.0001	7.23, 8.23	4.47, 5.73	< 0.0001
*IIEF- Orgasmic function*							
Placebo	Baseline	4.88 ± 1.02	-	-	-	-	-
	Day 14	5.1 ± 0.86	0.0459	-	-0.12, 0.56	-	-
	Day 42	5.85 ± 0.89	< 0.0001	-	0.62, 1.32	-	-
	Day 84	7.12 ± 0.91	< 0.0001	-	1.89, 2.59	-	-
LN18178	Baseline	4.67 ± 0.83		0.1491	-	-0.13, 0.55	-
	Day 14	5.77 ± 1.09	< 0.0001	< 0.0001	0.74, 1.46	0.31, 1.03	<0.0001
	Day 42	6.79 ± 0.77	< 0.0001	< 0.0001	1.82, 2.42	0.63, 1.25	<0.0001
	Day 84	8.81 ± 0.72	< 0.0001	< 0.0001	3.85, 4.43	1.39, 1.99	<0.0001
*IIEF- Sexual desire*							
Placebo	Baseline	3.71 ± 0.87	-	-	-	-	-
	Day 14	4.81 ± 1.14	< 0.0001	-	0.73, 1.47	-	
	Day 42	5.47 ± 0.92	< 0.0001	-	1.43, 2.09	-	-
	Day 84	6.64 ± 1	< 0.0001	-	2.59, 3.27	-	-
LN18178	Baseline	3.86 ± 0.77		0.2635	-	-0.15, 0.45	-
	Day 14	5.65 ± 0.81	< 0.0001	< 0.0001	1.50, 2.08	0.48, 1.20	0.0014
	Day 42	6.81 ± 0.67	< 0.0001	< 0.0001	2.68, 3.22	1.04, 1.64	< 0.0001
	Day 84	8.6 ± 0.88	< 0.0001	< 0.0001	4.43, 5.05	1.61, 2.30	< 0.0001
*IIEF- Intercourse satisfaction*							
Placebo	Baseline	6.97 ± 1.33	-	-	-	-	-
	Day 14	7.32 ± 1.29	0.0149		-0.13, 0.83	-	
	Day 42	8.85 ± 1.26	< 0.0001	-	1.41, 2.35	-	-
	Day 84	10.64 ± 1.24	< 0.0001	-	3.20, 4.14	-	-
LN18178	Baseline	6.6 ± 1.36		0.1237	-	-0.12, 0.86	-
	Day 14	8.25 ± 1.81	< 0.0001	< 0.0001	1.06, 2.24	0.35, 1.51	< 0.0001
	Day 42	10.33 ± 1.27	< 0.0001	< 0.0001	3.24, 4.22	1.01, 1.95	< 0.0001
	Day 84	12.74 ± 1.06	< 0.0001	< 0.0001	5.69, 6.59	1.68, 2.53	< 0.0001
*IIEF- Overall satisfaction*							
Placebo	Baseline	4.63 ± 0.98	-	-	-	-	-
	Day 14	5.1 ± 0.84	< 0.0001	-	0.14, 0.80	-	-
	Day 42	6.17 ± 0.85	< 0.0001	-	1.21, 1.87	-	-
	Day 84	6.8 ± 0.89	< 0.0001	-	1.83, 2.51	-	-
LN18178	Baseline	4.49 ± 1.05		0.3834	-	-0.23, 0.51	-
	Day 14	5.63 ± 1.14	< 0.0001	0.0004	0.73, 1.55	0.16, 0.90	0.0026
	Day 42	7.04 ± 0.73	< 0.0001	< 0.0001	2.21, 2.89	0.58, 1.16	< 0.0001
	Day 84	8.68 ± 0.74	< 0.0001	< 0.0001	3.85, 4.53	1.58, 2.18	< 0.0001
*IIEF- Total score*							
Placebo	Baseline	37.98 ± 3.07	-	-	-		-
	Day 14	40.56 ± 3.59	< 0.0001	-	1.36, 3.80		-
	Day 42	45.12 ± 3.13	< 0.0001	-	6.01, 8.27		-
	Day 84	51.64 ± 3.31	< 0.0001	-	12.50, 14.82		-
LN18178	Baseline	37.42 ± 2.66		0.2511		-0.5, 1.62	-
	Day 14	45.37 ± 4.74	< 0.0001	< 0.0001	6.52, 9.38	3.27, 6.35	< 0.0001
	Day 42	53.25 ± 3.33	< 0.0001	< 0.0001	14.71, 16.95	6.94, 9.32	< 0.0001
	Day 84	64.37 ± 3.09	< 0.0001	< 0.0001	25.88, 28.02	11.55, 13.91	< 0.0001
*EHS scores*							
Placebo	Baseline	1.97 ± 0.61	-	-			-
	Day 14	1.98 ± 0.68	0.9813	-	-0.23, 0.25		-
	Day 42	2.08 ± 0.6	0.2453		-0.11, 0.33		
	Day 84	2.12 ± 0.74	0.2233	-	-0.10, 0.40		-
LN18178	Baseline	2.02 ± 0.67		0.6632		-0.19, 0.29	-
	Day 14	2.47 ± 0.57	< 0.0001	< 0.0001	0.22, 0.68	0.26, 0.72	0.0079
	Day 42	3.12 ± 0.71	< 0.0001	< 0.0001	0.84, 1.36	0.80, 1.28	< 0.0001
	Day 84	3.65 ± 0.48	< 0.0001	<0.0001	1.41, 1.85	1.30, 1.76	< 0.0001

Values present mean ± SD. placebo (n=59) and LN18178 (n=57). CI: Confidence interval; P < 0.05 was considered as statistically significant for 'within the group' and 'between the groups' comparison analysis using paired t test and ANCOVA, respectively, as described in materials and methods.

**Table 4:** Assessment of Multidimensional Fatigue Inventory scores (MFI)

	Evaluation	Mean ± SD	P value (vs. baseline)	P value (vs. placebo)	95% CI (vs. Baseline)	95% CI (vs. Placebo)	P value, change from baseline (vs. placebo)
*General Fatigue*							
Placebo	Baseline	10.54 ± 2.12	-	-		-	-
	Day 42	10.68 ± 1.61	0.4063	-	-0.55, 0.83	-	-
	Day 84	10.58 ± 1.53	0.9458	-	-0.63, 0.71	-	-
LN18178	Baseline	11.23 ± 2.41		0.1058		-0.14, 1.52	-
	Day 42	11.02 ± 1.47	0.3739	0.5976	-0.53, 0.95	-0.22, 0.92	0.4170
	Day 84	9.58 ± 2.44	0.0020	0.054	0.75, 2.55	0.25, 1.75	0.0336
*Physical fatigue*							
Placebo	Baseline	9.03 ± 1.36	-	-		-	-
	Day 42	8.63 ± 1.27	0.0050	-	-0.08, 0.88	-	-
	Day 84	8.22 ± 1.39	0.0001	-	0.31, 1.31	-	-
LN18178	Baseline	8.86 ± 1.59		0.5267		-0.37, 0.71	-
	Day 42	8.4 ± 1.12	0.0254	0.6051	-0.05, 0.97	-0.21, 0.67	0.8792
	Day 84	6.47 ± 1.4	<0.000	<0.0001	1.83, 2.95	1.24, 2.26	<0.0001
*Reduced activity*							
Placebo	Baseline	8.36 ± 1.44	-	-		-	-
	Day 42	8.25 ± 1.21	0.6517	-	-0.38, 0.60	-	-
	Day 84	8.85 ± 1.49	0.1022	-	-0.04, 1.02	-	-
LN18178	Baseline	8.53 ± 1.38		0.5157		-0.35, 0.69	-
	Day 42	8.58 ± 1.13	0.8266	0.1663	-0.42, 0.52	-0.10, 0.76	0.5777
	Day 84	7.98 ± 2.56	0.1061	0.1022	-0.21, 1.31	0.10, 1.64	0.0954
*Reduced motivation*							
Placebo	Baseline	11.36 ± 1.84	-	-			-
	Day 42	11.25 ± 1.43	0.7078	-	-0.49, 0.71	-	-
	Day 84	11.86 ± 2.07	0.0773	-	-0.21, 1.21	-	-
LN18178	Baseline	11.7 ± 2.1		0.3460		-0.39, 1.07	-
	Day 42	11.11 ± 1.29	0.0410	0.3568	-0.06, 1.24	-0.36, 0.64	0.0783
	Day 84	11.16 ± 3.95	0.3367	0.4424	-0.63, 1.71	-0.45, 1.85	0.3295
*Mental fatigue*							
Placebo	Baseline	11.36 ± 1.7	-	-		-	-
	Day 42	11.32 ± 1.31	0.8442	-	-0.51, 0.59	-	-
	Day 84	10.56 ± 1.57	0.0039	-	0.20, 1.40	-	-
LN18178	Baseline	11.65 ± 2.07		0.4049		-0.41, 0.99	-
	Day 42	10.53 ± 1.38	< 0.0001	< 0.0001	0.47, 1.77	0.30, 1.28	0.0005
	Day 84	8.67 ± 1.52	< 0.0001	< 0.0001	2.31, 3.65	1.32, 2.46	< 0.0001
*Total score*							
Placebo	Baseline	56.51 ± 6.17	-	-		-	-
	Day 42	56.1 ± 3.74	0.4831	-	-1.45, 2.27	-	-
	Day 84	55.61 ± 5.12	0.4962	-	-1.17, 2.97	-	-
LN18178	Baseline	58.07 ± 8.07		0.2430		-1.08, 4.20	-
	Day 42	55.11 ± 3.94	0.0091	0.0100	0.60, 5.32	-0.42, 2.40	0.0877
	Day 84	50.09 ± 12.58	0.0009	0.3767	4.06, 11.90	2.01, 9.03	0.1866

Values present mean ± SD. placebo (n=59) and LN18178 (n=57). CI: Confidence interval; P < 0.05 was considered as statistically significant for 'within the group' and 'between the groups' comparison analysis using paired t-test and ANCOVA, respectively, as described in materials and methods.

**Table 5:** Assessment of PSQI scores

	Evaluation	Mean ± SD	P value (vs. baseline)	P value (vs. placebo)	95% CI vs. Baseline	95% CI vs. Placebo	P value, change from baseline (vs. placebo)
*Subjective sleep quality*							
Placebo	Baseline	1.66 ± 0.86	-	-	-	-	-
	Day 42	1.85 ± 0.94	0.1805	-	-0.14, 0.52	-	-
	Day 84	1.51 ± 0.97	0.4089	-	-0.18, 0.48	-	-
LN18178	Baseline	1.42 ± 0.80		0.1237	-	-0.07, 0.55	-
	Day 42	1.25 ± 0.79	0.2050	0.0015	-0.13, 0.47	0.28, 0.92	0.0693
	Day 84	0.72 ± 0.67	< 0.0001	< 0.0001	0.43, 0.97	0.48, 1.10	0.0160
*Sleep latency*							
Placebo	Baseline	2.22 ± 0.59	-	-	-	-	-
	Day 42	1.97 ± 0.56	0.0115	-	0.04, 0.46	-	-
	Day 84	1.71 ± 0.53	< 0.000	-	0.31, 0.71	-	-
LN18178	Baseline	2.18 ± 0.47		0.6510	-	-0.16, 0.24	-
	Day 42	1.96 ± 0.42	0.0075	0.8964	0.05, 0.39	-0.17, 0.19	0.5119
	Day 84	1.53 ± 0.50	< 0.0001	0.0763	0.47, 0.83	-0.01, 0.37	0.2870
*Sleep duration*							
Placebo	Baseline	0.83 ± 0.85	-	-		-	-
	Day 42	0.56 ± 0.68	0.0389	-	-0.01, 0.55	-	-
	Day 84	0.47 ± 0.63	0.0026	-	0.09, 0.63	-	-
LN18178	Baseline	0.47 ± 0.68		0.0147	-	0.08, 0.64	-
	Day 42	0.46 ± 0.71	0.8821	0.5223	-0.25, 0.27	-0.16, 0.36	0.1141
	Day 84	0.35 ± 0.64	0.3930	0.2960	-0.13, 0.37	-0.11, 0.35	0.1663
*Habitual sleep efficiency*							
Placebo	Baseline	0.15 ± 0.36	-	-	-	-	-
	Day 42	0.10 ± 0.30	0.5488	-	-0.07, 0.17	-	-
	Day 84	0.07 ± 0.25	0.2266	-	-0.03, 0.19	-	-
LN18178	Baseline	0.12 ± 0.38		0.6676	-	-0.11, 0.17	-
	Day 42	0.09 ± 0.29	0.7266	0.9276	-0.10, 0.16	-0.10, 0.12	0.8268
	Day 84	0.19 ± 0.48	0.4978	0.1131	-0.09, 0.23	-0.02, 0.26	0.1092
*Sleep disturbances*							
Placebo	Baseline	2.03 ± 0.26	-	-	-	-	-
	Day 42	1.62 ± 0.49	< 0.0001	-	0.27, 0.55	-	-
	Day 84	1.63 ± 0.49	< 0.0001	-	0.26, 0.54	-	-
LN18178	Baseline	2.00 ± 0.27		0.4904	-	-0.07, 0.13	-
	Day 42	1.77 ± 0.42	0.001	0.0849	0.10, 0.36	-0.02, 0.32	0.0683
	Day 84	1.28 ± 0.45	<0.0001	0.0002	0.58, 0.86	0.18, 0.52	0.0019
*Use of sleep medication*							
Placebo	Baseline	0.00 ± 0.00	-	-	-	-	-
	Day 42	0.00 ± 0.00	-	-	0, 0	-	-
	Day 84	0.00 ± 0.00	-	-	0, 0	-	-
LN18178	Baseline	0.00 ± 0.00	-	-	-	-	-
	Day 42	0.00 ± 0.00	-	-	0, 0	-	-
	Day 84	0.00 ± 0.00	-	-	0, 0	-	-
*Daytime dysfunction*							
Placebo	Baseline	1.46 ± 0.68	-	-	-	-	-
	Day 42	1.68 ± 0.68	0.0039	-	-0.03, 0.47	-	-
	Day 84	1.51 ±0.73	0.6592	-	-0.21, 0.31	-	-
LN18178	Baseline	1.70 ± 0.65		0.0509	-	-0.004, 0.48	-
	Day 42	1.12 ± 0.66	< 0.0001	< 0.0001	0.34, 0.82	0.31, 0.81	< 0.0001
	Day 84	1.05 ± 0.79	< 0.0001	0.0070	0.38, 0.92	0.18, 0.74	0.0003
*Global PSQI score*							
Placebo	Baseline	8.36 ± 1.81	-	^-^	-	-	-
	Day 42	7.78 ± 1.89	0.0096	-	-0.09, 1.25	-	-
	Day 84	6.90 ± 1.60	< 0.0001	-	0.84, 2.08	-	-
LN18178	Baseline	7.89 ± 1.71		0.1609	-	-0.18, 1.12	-
	Day 42	6.65 ± 1.60	< 0.0001	0.0009	0.63, 1.85	0.48, 1.78	0.0346
	Day 84	5.12 ± 1.69	< 0.0001	< 0.0001	2.14, 3.40	1.17, 2.39	0.0006

Values present mean ± SD. placebo (n=59) and LN18178 (n=57). CI: Confidence interval; P < 0.05 was considered as statistically significant for 'within the group' and 'between the groups' comparison analysis using paired t-test and ANCOVA, respectively, as described in materials and methods.

## Abbreviations

AAS: Anabolic-androgenic steroids
3β-HSD: 3β-Hydroxysteroid Dehydrogenase
ANCOVA: Analysis of Covariance
BMI: Body Mass Index
CI: Confidence Interval
CTRI: Clinical Trial Registry of India
CYP17A1: Cytochrome P450 family 17 subfamily A member 1
DISF-SR-M: Derogatis Interview for Sexual Functioning-Male
ED: Erectile Dysfunction
EHS: Erection Hardness Score
GCP: Good Clinical Practice
GHS: General Health Survey
HGS: hand-grip strength
ICH: International Conference on Harmonization
IEC: Independent Ethics Committee
IIEF: International Index of Erectile Function
MFI: Multi-dimensional Fatigue Inventory
PP: Per-Protocol
PSQI: Pittsburgh Sleep Quality Index
SD: Standard Deviation
SMWT: Six-Minute Walk Test
StAR: Steroid Acute Regulatory protein
TRT: Testosterone replacement therapy
US FDA: United States Food and Drug Administration
