# Supplementary material for: A Combination of Punica granatum Fruit Rind and Theobroma cacao Seed Extracts Enhances Sexual Function in Aging Males in a Randomized, Double-blind, Placebo-controlled Study
Source: Int J Med Sci. 2025 Jan 1;22(2):383–97. doi: 10.7150/ijms.99958 (PMC11704686; doi:10.7150/ijms.99958)
Supplement: Supplementary file 1 — Supplementary tables. [file ijmsv22p0383s1.pdf]

Supplementary Table S1: Inclusion-exclusion criteria

Inclusion criteria	Exclusion criteria
• Healthy and recreationally active males (Age: 40-70 years; BMI: 20-29 kg/m^2) with serum Total testosterone ≥ 300 ng/dL.	• Blood pressure >140/90 mmHg, fasting plasma glucose (FBG) >125 mg/dL and abnormal ECG.
• International index of erectile function (IIEF) score between 17 and 25. Sexual desire domain (Q.11 and Q.12) of IIEF score of < 3 each.	• Physical disability that may limit sexual function or erectile dysfunction.
• Stable sexual relationship and sexually active.	• Oligospermia or any other sexual problems
• Refrain from beginning any new energy-boosting, protein- or testosterone-boosting, or health-related therapies throughout the study and from taking vitamins, minerals, or herbal remedies for at least 15 days before the screening visit.	• History of any major/genital surgeries, psychiatric, endocrine, and sleep-related disorders, dysfunctions related to the genito-urinary system, including benign prostate hyperplasia, muscular dystrophy & cardiovascular diseases,
• Agreed to maintain current diet and activity level and refrain from drinking coffee, caffeinated drinks, or beverages 24 hrs before the evaluation days.	• Anabolic drugs, corticosteroids, PDE-5 inhibitors, and testosterone replacement therapy
• Willing to follow an approved birth control measure if partner has childbearing potential.	• Recreational drugs, alcohol (> 3 standard drinks per week), and smokers (> 3 cigarettes per day).
• Agreed to provide written informed consent.	• Participated in a clinical study within the last 30 days before recruitment or concurrently participating in another study.

Supplementary Table S2: Assessment of General health survey (GHS) scores

	Evaluation	Mean ± SD	P value (vs. baseline)	P value (vs. placebo)	95% CI (vs. Baseline)	95% CI (vs. placebo)	P value, change from baseline (vs. placebo)
Libido							
Placebo	Baseline	3.92 ± 1.039	-	-	-	-	-
	Day 14	3.85 ± 0.847	0.5922	-	-0.28, 0.42	-	-
	Day 42	3.95 ± 0.797	0.6717	-	-0.31, 0.37	-	-
	Day 84	3.92 ± 0.896	0.9611	-	-0.35, 0.35	-	-
LN18178	Baseline	3.74 ± 0.856		0.3158	-	-0.17, 0.53	-
	Day 14	4.33 ± 1.139	< 0.0001	< 0.0001	0.22, 0.96	0.11, 0.85	< 0.0001
	Day 42	4.98 ± 1.142	< 0.0001	< 0.0001	0.87, 1.61	0.67, 1.39	< 0.0001
	Day 84	5.53 ± 1.571	< 0.0001	< 0.0001	1.32, 2.26	1.14, 2.08	< 0.0001
Muscle mass							
Placebo	Baseline	3.85 ± 0.943	-	-	-	-	-
	Day 14	3.80 ± 0.886	0.6326	-	-0.28, 0.38	-	-
	Day 42	3.95 ± 0.818	0.3618	-	-0.22, 0.42	-	-
	Day 84	4.03 ± 0.850	0.1706	-	-0.15, 0.51	-	-
LN18178	Baseline	3.61 ± 0.901		0.1760	-	-0.10, 0.58	-
	Day 14	4.16 ± 0.841	< 0.0001	< 0.0001	0.23, 0.87	0.04, 0.68	< 0.0001
	Day 42	4.81 ± 1.109	< 0.0001	< 0.0001	0.83, 1.58	0.50, 1.22	< 0.0001
	Day 84	5.39 ± 1.532	< 0.0001	< 0.0001	1.31, 2.25	0.91, 1.81	< 0.0001
Muscle strength							
Placebo	Baseline	3.76 ± 1.023	-	-	-	-	-
	Day 14	3.83 ± 0.769	0.4765	-	-0.26, 0.40	-	-
	Day 42	3.90 ± 0.712	0.4090	-	-0.18, 0.46	-	-
	Day 84	4.05 ± 0.918	0.0221	-	-0.06, 0.64	-	-
LN18178	Baseline	3.77 ± 0.866		0.9584	-	0.31, 1.05	-
	Day 14	4.44 ± 1.000	< 0.0001	< 0.0001	0.32, 1.02	0.28, 0.94	< 0.0001
	Day 42	5.02 ± 1.142	< 0.0001	< 0.0001	0.87, 1.63	0.77, 1.47	< 0.0001
	Day 84	5.72 ± 1.612	< 0.0001	< 0.0001	1.47, 2.43	1.19, 2.15	< 0.0001
Energy							
Placebo	Baseline	4.20 ± 0.924	-	-	-	-	-
	Day 14	4.22 ± 0.832	0.9528		-0.30, 0.34	-	-
	Day 42	4.07 ± 0.848	0.3603	-	-0.19, 0.45	-	-
	Day 84	4.27 ± 0.887	0.7083	-	-0.26, 0.40	-	-
LN18178	Baseline	3.74 ± 0.791		0.0043	-	0.14, 0.78	-
	Day 14	4.39 ± 0.774	< 0.0001	0.0018	0.36, 0.94	-0.13, 0.47	< 0.0001
	Day 42	4.95 ± 1.109	< 0.0001	< 0.0001	0.85, 1.57	0.52, 1.24	< 0.0001
	Day 84	5.77 ± 1.402	< 0.0001	< 0.0001	1.61, 2.45	1.07, 1.93	< 0.0001
Stamina							
Placebo	Baseline	3.75 ± 1.044	-	-	-	-	-
	Day 14	3.81 ± 0.819	0.4765	-	-0.28, 0.40	-	-
	Day 42	3.88 ± 0.697	0.3263	-	-0.19, 0.45	-	-
	Day 84	4.14 ± 0.918	0.0066	-	0.03, 0.75	-	-
LN18178	Baseline	3.54 ± 0.803		0.2466	-	-0.13, 0.55	-
	Day 14	4.11 ± 0.838	< 0.0001	0.0004	0.27, 0.87	-0.005, 0.60	0.0004
	Day 42	4.93 ± 1.067	< 0.0001	< 0.0001	1.04, 1.74	0.72, 1.38	< 0.0001
	Day 84	5.51 ± 1.594	< 0.0001	< 0.0001	1.50, 2.44	0.89, 1.85	< 0.0001
Sleep							
Placebo	Baseline	3.69 ± 0.987	-	-	-	-	-

	Day 14	4.02 ± 0.881	0.0042	-	-0.01, 0.67	-	-
	Day 42	4.32 ± 0.973	0.0004	-	0.27, 0.99	-	-
	Day 84	4.53 ± 1.056	< 0.0001	-	0.47, 1.21	-	-
LN18178	Baseline	3.70 ± 0.823		0.9678	-	-0.32, 0.34	-
	Day 14	4.54 ± 0.908	< 0.0001	0.0005	0.52, 1.16	0.19, 0.85	0.0011
	Day 42	5.37 ± 0.938	< 0.0001	< 0.0001	1.34, 2.00	0.70, 1.40	< 0.0001
	Day 84	6.30 ± 1.133	< 0.0001	< 0.0001	2.23, 2.97	1.37, 2.17	< 0.0001
Total GHS score							
Placebo	Baseline	23.17 ± 3.649	-	-	-	-	-
	Day 14	23.53 ± 2.812	0.2248	-	-0.83, 1.55	-	-
	Day 42	24.07 ± 3.090	0.1220	-	-0.33, 2.13	-	-
	Day 84	24.93 ± 3.908	0.0005	-	0.38, 3.14	-	-
LN18178	Baseline	22.11 ± 2.920		0.9678	-	-0.16, 2.28	-
	Day 14	25.96 ± 3.756	< 0.0001	< 0.0001	2.60, 5.10	1.21, 3.65	< 0.0001
	Day 42	30.05 ± 4.454	< 0.0001	< 0.0001	6.54, 9.34	4.57, 7.39	< 0.0001
	Day 84	34.21 ± 7.837	< 0.0001	< 0.0001	9.91, 14.29	7.01, 11.55	< 0.0001

Values present mean ± SD. placebo (n=59) and LN18178 (n=57). CI: Confidence interval; P < 0.05 was considered as statistically significant for 'within the group' and 'between the groups' comparison analysis using paired t-test and ANCOVA, respectively, as described in materials and methods

Supplementary Table S3: Adverse events

Group	Adverse events	Severity
Placebo (n=59)	Bloating (1)	Mild
	Abdominal pain (1)	Mild
LN18178 (n=57)	Vomiting (1)	Mild
	Nausea (1)	Mild
	Headache (1)	Mild

Supplementary Table S4: Safety parameters

Parameters	Group	n	Mean ± SD	Change from baseline (Mean ± SD)	P value (vs. baseline)	P value (vs. placebo)
			Hematology			
Hemoglobin (g/dL)						
Baseline	Placebo	60	14.66 ± 0.94	-		
	LN18178	60	14.38 ± 0.87	-		0.0955
Day 84	Placebo	59	14.55 ± 0.69	-0.09 ± 1.06	0.5112	
	LN18178	57	14.46 ± 0.67	0.06 ±1.07	0.6937	0.4626
Platelet count (10^5/µL)						
Baseline	Placebo	60	253.05 ± 56.99	-		
	LN18178	60	255.05 ± 66.20	-		0.8595
Day 84	Placebo	59	248.81 ± 52.51	-2.42 ± 73.17	0.8001	
	LN18178	57	254.67 ± 40.26	0.26 ± 70.48	0.9776	0.5029
Red blood cells (10^6/mm^3)						
Baseline	Placebo	60	4.97 ± 0.30	-		
	LN18178	60	4.90 ± 0.30	-		0.2505
Day 84	Placebo	59	4.99 ± 0.23	0.02 ± 0.32	0.5733	
	LN18178	57	5.06 ± 0.30	0.15 ± 0.34	0.0013	0.113
Erythrocyte sedimentation rate (mm/hr)						
Baseline	Placebo	60	6.08 ± 2.08	-		0.9657
	LN18178	60	6.10 ± 2.16	-		
Day 84	Placebo	59	6.51 ± 1.82	0.39 ± 2.41	0.2198	0.7354
	LN18178	57	6.10 ± 2.16	0.19 ± 2.79	0.6032	
Hematocrit (%)						
Baseline	Placebo	60	44.92 ± 2.89			0.76
	LN18178	60	45.07 ± 2.40			
Day 84	Placebo	59	45.08 ± 2.58	0.22 ± 3.47	0.6326	0.3032
	LN18178	57	45.58 ± 2.61	0.55 ± 2.88	0.1538	
Total white blood cells count (10^3/µL)						
Baseline	Placebo	60	7.08 ± 1.43	-		0.4797
	LN18178	60	7.27 ± 1.51	-		
Day 84	Placebo	59	7.81 ± 1.21	0.70 ± 1.48	0.0006	0.2297
	LN18178	57	8.08 ± 1.17	0.82 ± 1.65	0.0004	
Neutrophils (%)						
Baseline	Placebo	60	60.33 ± 6.01	-		0.1447
	LN18178	60	61.97 ± 6.18	-		
Day 84	Placebo	59	63.61 ± 6.02	3.22 ± 6.82	0.0006	0.6515
	LN18178	57	64.11 ± 5.74	1.89 ± 6.58	0.034	
Lymphocytes (%)						
Baseline	Placebo	60	30.18 ± 4.82	-		0.2958
	LN18178	60	29.25 ± 4.91	-		
Day 84	Placebo	59	28.14 ± 5.12	-2.08 ± 6.02	0.0100	0.6731
	LN18178	57	27.74 ± 5.03	-1.33 ± 5.72	0.0841	
Eosinophils (%)						
Baseline	Placebo	60	2.90 ± 1.31	-		0.0234
	LN18178	60	2.40 ± 1.06	-		
Day 84	Placebo	59	2.78 ± 1.04	-0.07 ± 1.65	0.7533	0.4119
	LN18178	57	2.61 ± 1.13	0.28 ± 1.53	0.1722	
Monocytes (%)						
Baseline	Placebo	60	6.58 ± 1.88	-		0.4912

	LN18178	60	6.35 ± 1.82	-		
Day 84	Placebo	59	5.47 ± 1.51	-1.07 ± 2.10	0.0002	
	LN18178	57	5.54 ± 1.64	-0.81 ± 2.08	0.0050	0.8132
Basophils (%)						
Baseline	Placebo	60	0.00 ± 0.00	-		1.000
	LN18178	60	0.00 ± 0.00	-		
Day 84	Placebo	59	0.00 ± 0.00	0.00 ± 0.00	1.000	1.000
	LN18178	57	0.00 ± 0.00	0.00 ± 0.00	1.000	
Blood biochemistry						
Fasting Blood Glucose (mg/dL)						
Baseline	Placebo	60	87.65 ± 6.16	-		0.9672
	LN18178	60	87.60 ± 7.08	-		
Day 84	Placebo	59	89.71 ± 5.49	1.90 ± 7.96	0.0722	0.3156
	LN18178	57	88.75 ± 4.69	1.07 ± 7.36	0.2770	
Creatinine (mg/dL)						
Baseline	Placebo	60	0.92 ± 0.12	-		0.7239
	LN18178	60	0.92 ± 0.13	-		
Day 84	Placebo	59	0.90 ± 0.11	-0.01 ± 0.16	0.5294	0.5290
	LN18178	57	0.92 ± 0.16	0.00 ± 0.20	0.9217	
Blood urea nitrogen (mg/dL)						
Baseline	Placebo	60	15.51 ± 3.29	-		0.6600
	LN18178	60	15.75 ± 2.63	-		
Day 84	Placebo	59	16.32 ± 2.10	0.80 ± 3.37	0.0724	0.2580
	LN18178	57	15.84 ± 2.49	0.19 ± 2.99	0.6283	
Uric Acid (mg/dL)						
Baseline	Placebo	60	5.72 ± 0.82	-		0.3248
	LN18178	60	5.57 ± 0.81	-		
Day 84	Placebo	59	5.69 ± 0.79	0.00 ± 0.97	0.9787	0.0416
	LN18178	57	5.98 ± 0.70	0.44 ± 1.03	0.0023	
Sodium (mmol/L)						
Baseline	Placebo	60	139.87 ± 2.30	-		0.8761
	LN18178	60	139.93 ± 2.38	-		
Day 84	Placebo	59	140.07 ± 2.26	0.19 ± 3.23	0.6596	0.3129
	LN18178	57	140.47 ± 2.05	0.58 ± 3.15	0.1708	
Potassium (mmol/L)						
Baseline	Placebo	60	4.66 ± 0.32	-		0.4258
	LN18178	60	4.70 ± 0.34	-		
Day 84	Placebo	59	4.51 ± 0.43	-0.14 ± 0.43	0.0138	0.9413
	LN18178	57	4.52 ± 0.39	-0.20 ± 0.43	0.0012	
Alkaline Phosphate (IU/L)						
Baseline	Placebo	60	90.57 ± 21.79	-		0.9898
	LN18178	60	90.62 ± 21.08	-		
Day 84	Placebo	59	92.53 ± 20.38	1.20 ± 16.10	0.5681	0.8260
	LN18178	57	93.37 ± 20.82	2.51 ± 10.83	0.0858	
Aspartate transaminase (U/L)						
Baseline	Placebo	60	22.87 ± 5.52	-		0.5675
	LN18178	60	22.28 ± 5.62	-		
Day 84	Placebo	59	23.95 ± 4.68	1.00± 6.21	0.2214	0.5145
	LN18178	57	24.51 ± 4.53	2.19 ±6.85	0.0190	
Alanine aminotransferase (U/L)						
Baseline	Placebo	60	30.78 ± 8.23	-		0.1682

	LN18178	60	28.80 ± 7.42	-		
Day 84	Placebo	59	34.19 ± 7.79	3.34 ±9.32	0.0079	0.8501
	LN18178	57	33.88 ± 9.72	33.88 ± 9.72	0.0001	
Bilirubin (mg/dL)						
Baseline	Placebo	60	0.63 ± 0.21	-		0.6104
	LN18178	60	0.61 ± 0.19	-		
Day 84	Placebo	59	0.68 ± 0.20	0.05 ± 0.31	0.2552	0.6602
	LN18178	57	0.66 ± 0.21	0.04 ± 0.29	0.3246	
Albumin (gm/dL)						
Baseline	Placebo	60	4.39 ± 0.31	-		0.3020
	LN18178	60	4.33 ± 0.31	-		
Day 84	Placebo	59	4.33 ± 0.36	-0.06 ± 0.51	0.4066	0.2805
	LN18178	57	4.33 ± 0.31	-0.07 ± 0.48	0.2641	
Low-density lipoprotein (mg/dL)						
Baseline	Placebo	60	75.22 ± 17.62	-		0.1514
	LN18178	60	79.57 ± 15.25	-		
Day 84	Placebo	59	83.48 ± 10.80	7.93 ± 19.10	0.0023	0.6105
	LN18178	57	84.49 ± 10.59	5.28 ± 17.22	0.0244	
High-density lipoprotein (mg/dL)						
Baseline	Placebo	60	46.45 ± 5.41	-		0.0683
	LN18178	60	48.41 ± 6.21	-		
Day 84	Placebo	59	48.33 ± 6.14	1.77 ± 7.01	0.0566	0.2507
	LN18178	57	49.67 ± 6.37	1.80 ± 8.57	0.1182	
Very-low-density lipoprotein (mg/dL)						
Baseline	Placebo	60	22.66 ± 5.84	-		0.8283
	LN18178	60	22.43 ± 5.59	-		
Day 84	Placebo	59	24.40 ± 3.45	1.83 ± 6.53	0.0351	0.8762
	LN18178	57	24.29 ±3.92	1.96 ± 5.98	0.0163	
Triglycerides (mg/dL)						
Baseline	Placebo	60	120.66 ±19.09	-		0.3658
	LN18178	60	123.76 ± 18.31	-		
Day 84	Placebo	59	129.01 ± 14.77	8.64 ± 18.81	0.0008	0.3890
	LN18178	57	131.25 ± 13.10	8.30 ± 16.92	0.0005	
Total cholesterol (mg/dL)						
Baseline	Placebo	60	144.33 ±18.62	-		0.0743
	LN18178	60	150.41 ± 18.33	-		
Day 84	Placebo	59	156.22 ± 13.62	11.55 ± 23.17	0.0003	0.3624
	LN18178	57	158.46 ±12.76	9.04 ± 20.99	0.002	
Creatine Kinase (U/L)						
Baseline	Placebo	60	81.60 ± 11.44	-	-	0.8981
	LN18178	60	81.33 ± 11.31	-	-	
Day 84	Placebo	59	82.19 ± 9.97	-0.59 ± 1.47	0.5499	< 0.0001
	LN18178	57	74.07 ± 9.07	7.26 ± 2.24	< 0.0001	

Values present mean ± SD. P < 0.05 was considered as statistically significant for 'within the group' and 'between the groups' comparison analysis using paired t-test and ANCOVA, respectively.
